# Genetic Evidence of the Black Death in the Abbey of San Leonardo (Apulia Region, Italy): Tracing the Cause of Death in Two Individuals Buried with Coins

**DOI:** 10.3390/pathogens10111354

**Published:** 2021-10-20

**Authors:** Donato Antonio Raele, Ginevra Panzarino, Giuseppe Sarcinelli, Maria Assunta Cafiero, Anna Maria Tunzi, Elena Dellù

**Affiliations:** 1 Istituto Zooprofilattico Sperimentale della Puglia e della Basilicata, 71121 Foggia, Italy; mariaassunta.cafiero@izspb.it; 2 Departament de Prehistòria i Arqueologia, Universitat de Valéncia, 46003 Valencia, Spain; ginevrapanzarino@gmail.com; 3 Department of Cultural Heritage, Università del Salento, 73100 Lecce, Italy; giuseppe.sarcinelli@unisalento.it; 4 Soprintendenza ABAP per la Città Metropolitana di Bari, Laboratory of Physical Anthropology, 70121 Bari, Italy; annamaria.tunzi@beniculturali.it (A.M.T.); elena.dellu@beniculturali.it (E.D.)

**Keywords:** *Yersinia pestis*, plague, Black Death, coins, ancient DNA, Italy

## Abstract

The Abbey of San Leonardo in Siponto (Apulia, Southern Italy) was an important religious and medical center during the Middle Ages. It was a crossroads for pilgrims heading along the Via Francigena to the Sanctuary of Monte Sant’Angelo and for merchants passing through the harbor of Manfredonia. A recent excavation of Soprintendenza Archeologica della Puglia investigated a portion of the related cemetery, confirming its chronology to be between the end of the 13th and beginning of the 14th century. Two single graves preserved individuals accompanied by numerous coins dating back to the 14th century, hidden in clothes and in a bag tied to the waist. The human remains of the individuals were analyzed in the Laboratorio di Antropologia Fisica of Soprintendenza ABAP della città metropolitana di Bari. Three teeth from each individual were collected and sent to the Istituto Zooprofilattico Sperimentale di Puglia e Basilicata to study infectious diseases such as malaria, plague, tuberculosis, epidemic typhus and Maltese fever (Brucellosis), potentially related to the lack of inspection of the bodies during burial procedures. DNA extracted from six collected teeth and two additional unrelated human teeth (negative controls) were analyzed using PCR to verify the presence of human DNA (β-globulin) and of pathogens such as *Plasmodium* spp., *Yersinia pestis*, *Mycobacterium* spp., *Rickettsia* spp. and *Brucella* spp. The nucleotide sequence of the amplicon was determined to confirm the results. Human DNA was successfully amplified from all eight dental extracts and two different genes of *Y. pestis* were amplified and sequenced in 4 out of the 6 teeth. Molecular analyses ascertained that the individuals buried in San Leonardo were victims of the Black Death (1347–1353) and the data confirmed the lack of inspection of the corpses despite the presence of numerous coins. This study represents molecular evidence, for the first time, of Southern Italy’s involvement in the second wave of the plague pandemic.

## 1. Introduction

**Figure 1:**
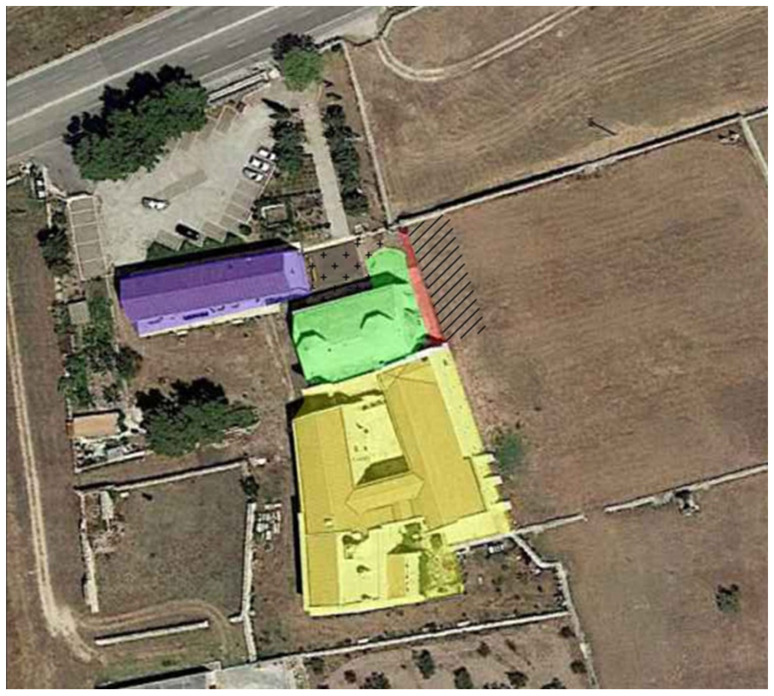
Abbey of San Leonardo: the church (green), the hospital (purple) and the monastery (yellow); the area of the archaeological investigation (red), the area where the cemetery was believed to be located [12] (crosses); the area where the cemetery continues to develop (oblique lines) [photo Google Earth 2019].

**Figure 2:**
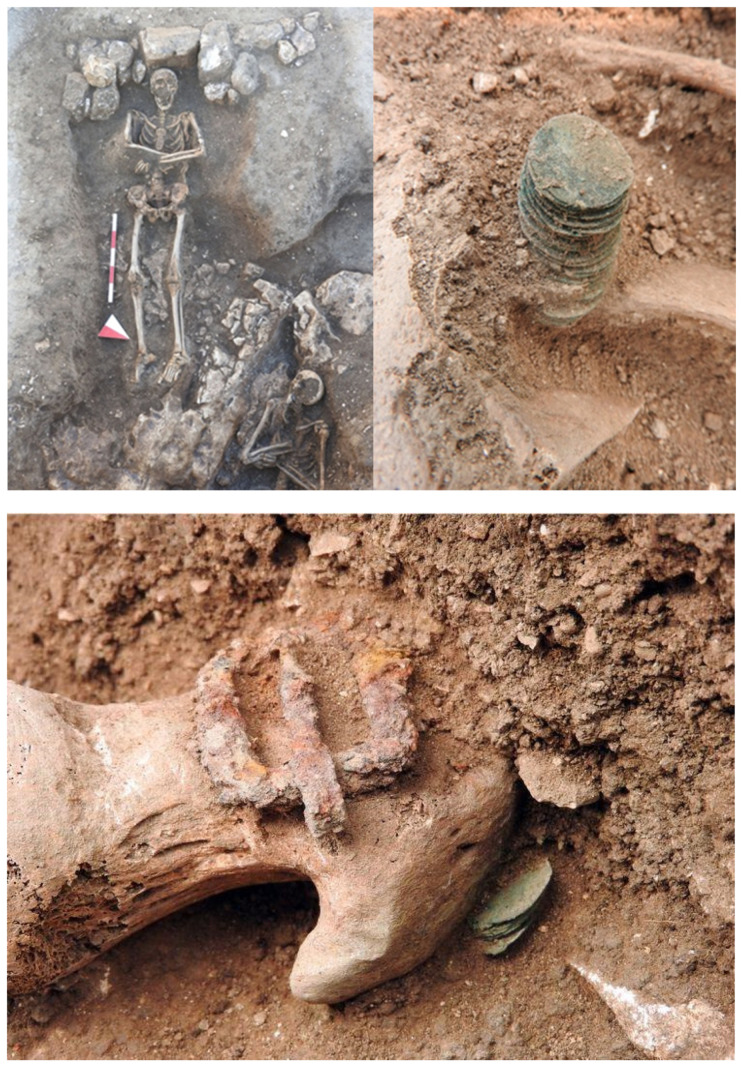
Abbey of San Leonardo: Details of burial site and coins with the human remains.

The Abbey of San Leonardo di Siponto in Lama Volara is an important religious and medical center near Manfredonia (Foggia) in Southern Italy [1,2,3,4,5]. The church, founded at the beginning of the 12th century, became an important property belonging to the Teutonic Knights of St. Mary of Jerusalem from 1260 to 1480 [6]. During the Middle Ages, along the Via Peregrinorum, numerous *hospitia* and *xenodochia* arose to shelter travelers and were entrusted to the management of religious and knightly orders [7]. The Abbey of San Leonardo was one such shelter, a very important crossroad for pilgrims heading along the Via Francigena to the Sanctuary of Monte Sant’Angelo and for merchants passing through the harbor of Siponto [8]. Pilgrims, the poor and the sick (including merchants, travelers or farmers) were welcomed in the hospital and cared for. [9]. The privileged role of Siponto is due to the city’s position with respect to the progressive road system already structured during the Roman period. Unfortunately, the strategic position of this important religious and medical center contrasted with the geomorphology of the territory, which up until the 1900s was a disease-ridden swamp. These environmental and climatic conditions caused the spread of diseases such as malaria, widespread in the area. Almost 50% of hospitalized patients in the summer and autumn months were referred to as Malaria cases [10]. In 2015, archaeological investigations carried out by Soprintendenza Archeologica della Puglia (directed by Dr. A.M. Tunzi) allowed for investigating a small portion of the cemetery area outside the church. In fact, on the northern side, behind the apses and the chapel of San Leonardo, the funerary area was developed [10,11]. The area was closed on the northern side by a wall built during the Teutonic period (the end of thirteenth–early fourteenth century) [12]. A total of twenty tombs were discovered, shown in Figure 1. Two of them (Graves 2 and 6, here named “T.2” and “T.6”) were different from the others, a hoard of coins was discovered in these two graves [13] (Figure 2). The data allowed us to develop interesting hypotheses on their state of health at the time of death. The most suggestive theory, based on the chronology of the tombs, was that they had died from an infectious disease. For this reason, anthropological, paleopathological, and, above all, molecular analyses were carried out.

## 2. Materials and Methods

### 2.1. The Graves: Bones, Biological Profiles and Macroscopic Paleopathology

The biological and paleopathological profiles of individuals have been reconstructed according to the following aspects: identification of metric and morphometric features [14,15,16]; determination of age and sex [17,18,19,20,21,22,23,24]; identification of nutritional and/or stress diseases, periodontal diseases and caries [19,25]; markers of skeletal biomechanical stress—syndesmosis lesions, enthesopathies, new articular surfaces and degenerative joint diseases [26,27,28,29]; paleopathological conditions [30,31,32].

### 2.2. The Graves: Archaeological, Taphonomic and Numismatic Data

The material refers to two individuals. The T.2 skeleton is not complete and is preserved up to the pelvis, while that of T.6 is complete, although the tomb was identified by excavating a planting pit (Figure 2). The state of conservation is not excellent: in both cases, the bones are fragile, with numerous cracks, chromatic alterations, and partial bleaching on the cortices. These conditions are due to the diagenesis in dry and clay soil and due to the presence of root systems that have plants affecting the surfaces, weakening them, and making them decompose due to evaporation processes [33,34,35]. At the same time, the tombs were superficial and damaged by recent work in the Abbey. For these reasons, the bones were packed with paper during the excavation to avoid losing the smallest fragments during transport and were cleaned in the laboratory according to the most modern conservation methods for human remains [36,37,38,39]. The funerary and taphonomic aspects are classified according to Duday’s definitions [40,41].

The coins, aggregated together and concretized, were washed in an ammonia and alcohol solution (1/10), then they were separated manually and cleaned using a scalpel. The identification and dating of the coins were carried out using the descriptions of D.M. Metcalf, J. Baker, E. Biaggi, P.H. Grierson, L. Travaini, and the catalogue of Italian coins in the British Museum [42,43,44,45,46].

### 2.3. Ancient DNA (aDNA): Extraction, Procedures and Analysis

Dental samples were collected from human remains belonging to two distinct individuals, named T.2 and T.6, buried in separate tumuli along the external wall perimeter of the Abbey of San Leonardo in Siponto. Specifically, the anthropologists collected one molar, one premolar, and one incisor directly from the maxillary bone of each of the two individuals. In addition, a further dental sample was collected from the remains of another individual whose death was not related to infectious causes. All samples were collected using sterile tools, and each sampling was carried out with different devices. Immediately after collection, the samples were placed singularly in sterile vessels and sent to the facilities of the Istituto Zooprofilattico Sperimentale della Puglia e della Basilicata (IZSPB). As recommended by Parson and Weedn [47], for the manipulation of samples at high risk of contamination, four different laboratories were selected (LB1, LB2, LB3, and LB4). Each laboratory works independently, with dedicated staff, equipment, and reagents, and are distant from each other from a few meters to several hundred km. The preliminary operations were carried out in LB1. Specifically, each tooth was placed in a 25 mL sterile gamma-irradiated tube and washed with 10 mL of PBS. Each tooth underwent three PBS washes, each in a sterile tube. After washing, the samples were laid upon an aluminum layer and were UV irradiated in a shielded chamber for 24 h. After sterilization of the external faces, the teeth were longitudinally sectioned by using a sterile diamond knife. The pulpal material was removed and collected using a sterile probe in a 1.5 mL sterile tube and stored at –20 °C.

The sample preparation laboratory (LB1) is located in the Medical Clinic of Dr. Luigi Ciuffreda in Manfredonia (FG), approximately 42 km from the main laboratory; personal protective equipment (shirts, gloves, masks, protective glasses, and caps) and instruments (diamond cutter, mirrors, and containers) were sterilized and cleaned.

The aDNA extraction laboratory (LB2) is located in IZSPB in Foggia (S.S. Research and Development); in LB2, DNA of the targets investigated by this study has never been extracted and/or processed. The staff are dedicated, and the instruments consist of BL2 with a laminar flow hood, thermostat, centrifuge, tubes, tips, and sterile micropipettes. All reagents were reconstituted and used for the first time.

The purification of the total genomic DNA was carried out using a PrepFiler BTA Forensic DNA Extraction Kit (Thermo Scientific, Milan, Italy) in LB2. Each sample underwent DNA extraction alone, and two negative extraction controls, consisting of sterile water, were included in each purification procedure.

The laboratory chosen for the amplification and purification of aDNA (LB3) is located in IZSPB in Foggia (S.S. Virology). In LB3, DNA objects of this investigation were never extracted and/or processed; the equipment included BL2 with a laminar flow hood, thermostat, centrifuge, tubes, tips, and sterile micropipettes. Reagents and solutions were reconstituted and used for the first time without positive controls according to the “suicide-qPCR” method [48,49].

All DNA solutions were kept frozen at –20 °C and thawed immediately before PCR. Specifically, suicide-PCR techniques were carried out for the detection of *Brucella* spp. [50], *Rickettsia* spp. [51], *Mycobacterium tuberculosis* complex [52], *Bartonella* spp. [53], *Yersinia pestis* [54], *Plasmodium* spp. [55], using primers and probes previously described (Table 1). To prevent potential contamination, no positive control was used for pathogens. A RT-qPCR was performed to verify the presence of human DNA, targeting the *β-globin* gene [56]. Negative controls with sterile distilled water and elution buffer were included. When positive reactions were observed, the PCR products were purified using a GeneJET PCR Purification Kit (Thermo Scientific) and stored at –20 °C.

To confirm a positive plague result, a second amplified and sequenced target was analyzed. A suicide-Nested PCR targeting the *Y. pestis glpD* gene, encoding glycerol-3-phosphate dehydrogenase, was carried out according to a previously published assay [48].

The sequencing laboratory of the amplicons obtained (LB4) is the Sequencing Collection Milano (Eurofinsgenomics) located in Vimodrone (MI). The nucleotide sequences of the amplicons were determined by the Sanger method using the BigDye Terminator system at the facilities of Eurofins Genomics (LB4). Then, the nucleotide sequences were assembled by using CAP3 [57] and submitted to the GenBank after the removal of the primers. Finally, the sequences were compared with those present in the GenBank non-redundant nucleotide database by BLAST.

## 3. Results

### 3.1. Anthropological and Paleopathological Analysis

Individual T.2 was preserved exclusively for the skull and the upper portion of the post-cranium, including the pelvis. On a morphological and anthropometric level, the discriminating districts make it possible to identify the skeleton as a male subject of between 30 and 40 years of age and approximately 172 ± 4 cm in height.

The individual’s bone remains were subjected to a specific analysis to identify the most stressed joints. No asymmetrical relationship between the right and left sides of the shoulder was observed; the insertion of the coracoclavicular ligament and the subclavian and trapezius muscles being almost similar and with slight stresses; the same situation can be traced back to the origins of the pectoralis major and sternohyoid muscles. Moderate stresses are identified at the insertion of the pectoral, grand dorsal, and grand teres muscles and at the origins of the common tendon of the flexor and extensor muscles. The radii and ulna show symmetrical stresses of mild intensity both at the origins and insertions of the main muscles. The anterior parietal and frontal bones of the skull show intracranial granulations of Pacchioni. At the mandibular level, there is a marked periodontal disease (retraction of the alveolar margin and destructive caries in the right M3). The upper portion of the skeleton shows a primary OA localized exclusively in some areas of the post-cranium, mainly of a neuromechanical type and connected with the subject’s age. There is a quadrangular hole on the skull with non-remodeled margins (8 × 10 mm) and laceration at the intracranial level, which could be attributable to a perimortem trauma.

It possible to identify the individual T6 as a male subject of between 40 and 50 years of age and approximately 171 ± 4 cm in height.

At the scapular level, medium intensity enthesis is observed at the origin of the deltoid and latissimus dorsi (both strongly OA) at the insertion of the trapezius muscle, the great rhomboid, and the great dentate. The lower joints show symmetry and predominance concerning the superior appendicular skeleton. A strong enthesis is observed upon the insertion of the gluteus maximus muscle (OA beaks), comb, and adductor major. The pathological analysis shows a slight diffuse hyperostosis on the skull on both external parietals (diffuse microporosity) and some granulations of Pacchioni in the anterior and internal parietals. At the mandibular level, a marked periodontal disease is observed (retraction of the alveolar margin and antemortem loss of the right PM3). The individual shows a widespread primary OA both in the skull and in the post-cranium, mainly of a neuromechanical type and connected with the subject’s advanced age.

Of particular interest are two pathological lesions, likely to be due to an infectious illness, found in the left scapular fossa and in the right iliac fossa. In the first case, there is a centimetric bone loss (width 10 mm and length 45 mm) along the lateral margin of the scapula, near the origin of the brachial triceps muscle (long head) and the teres minor. In this case, and in the right pelvis, diffuse pitting and an irregularity of the bone surface are observed, with the presence of lamellar plates of newly formed bone on the surface and abscess cavities. In addition, cloacae are observed both in the fracture section of the bone and in the upper–lower norm and have a channel-passing course between involucrum and sequestrum. The iliac fossa does not show tissue loss but exclusive bone-repairing growth with a semicircular morphology; this starts almost in the proximity of the crest to proceed in the direction of the arched line, stopping just before it and, therefore, at approximately 2/3 of the wing. Along this semicircular edge, cavities with circular termination and channeled development between *involucrum* and *sequestrum* can be observed.

### 3.2. Archaeological, Taphonomic and Numismatic Data

The investigated cemetery is composed of 20 tombs related to 2 burial sessions, very close in age, both referable to the Late Medieval period, according to the chronology attested by written sources [58]. The graves are single burials dug into the bedrock or in the ground, rectangular and oval-shaped. The individuals are buried in the supine position, with heads to the west, arms flexed or crossed over the chest, and legs extended and parallel. The graves T.2 and T.6 are both located in the northern area of the cemetery. As for the relative chronology, T.2 refers to the first and oldest depositional phase. T.6, on the other hand, which is more superficial, refers to the second and last depositional phases. T.2 is carved into the bedrock, while T.6 is rectangular-shaped and was dug into the ground, with the part of the head limited by large blocks and stones. The bodies are both in primary position in anatomical connection. The first from T.2 is an adult male skeleton that is preserved from the pelvis up. The man wore a belt with a rectangular iron buckle with a shaft, found flattened on his right femur. Maybe tied to this belt was a small bag that contained 12 coins found stacked under the leg. There are 12 denarii in a silver mixture, strongly concentrated in issue in Ancona towards the half of the thirteenth century and Ravenna between 1232 and the fourteenth century. The second body, from T.6—also an adult male—was placed supine with the head to the west, the arms crossed over the abdomen, and the legs extended and parallel. The man guarded 99 alloy Deniers tournois from Frankish Greece (last decades of the 13th century to the first quarter of the 14th century) and one silver Gigliato issued in the name of Robert of Anjou (1309–1343), found in piles, probably inside bags hidden in various parts of his clothes. Three Deniers were placed on the pelvis, one of which was recovered with fragments of cloth. During their excavation, the coins appeared concreted together, keeping the shape of the three piles in which they were subdivided, and arranged as follows:

- One on the right hemithorax near the sternum: 39 Deniers;
- One right above the clavicle near the right shoulder: 14 Deniers;
- One under the humerus and close to the right scapula: 43 Deniers and the Gigliato.

Instead, the three Deniers found on the iliac crest of the left coxal bone of the pelvis were kept in a pocket as available cash for immediate expenses, while the rest of the hoard remained well concealed, sewn inside the garments. Whoever hid the coins did not divide the three sums into equal numbers, in whole quantities, or ordered by issuing authorities. The oldest Deniers tournois were issued by William of Villehardouin, 1246–1278, and the most recent was issued by John of Gravina, 1322–1333. The Gigliato, in “poor” style, with the inscription “ROBERT DEI GRA IERL ET SCIL’ RE”, is attributable to Robert of Anjou (1309–1343) but must be a posthumous emission.

### 3.3. Molecular Analysis

Human DNA was successfully amplified from all seven dental extracts. Among the pathogen-specific qPCR, the *pla* gene of *Y. pestis* was amplified in four samples, specifically, two premolars and two incisors from T.2 and T.6. No other amplification was detected from the other samples, including the extraction and qPCR negative controls.

In addition, the amplification of *glpD* gene of *Y. pestis* yielded an amplicon of 288-bp from two samples, one premolar and one incisor from T.2.and T.6. respectively.

The nucleotide sequences of the amplification products of the positive samples were 100% identical to the corresponding portion of the *pla* gene harbored by the plasmid of *Y. pestis* (reference sequence NC_017170). The partial sequence of the *pla* gene was submitted to GenBank under the accession number MW618095 (Figure S2).

The analysis of the *glpD* gene sequences revealed a similarity of 100% with that of the *Y. pestis* (AY312360, AF377937, and DQ073797). The nucleotide sequence of the DNA segment containing the *glpD* gene was submitted to GenBank under the accession number OK169606.

## 4. Discussion

**Figure 3:**
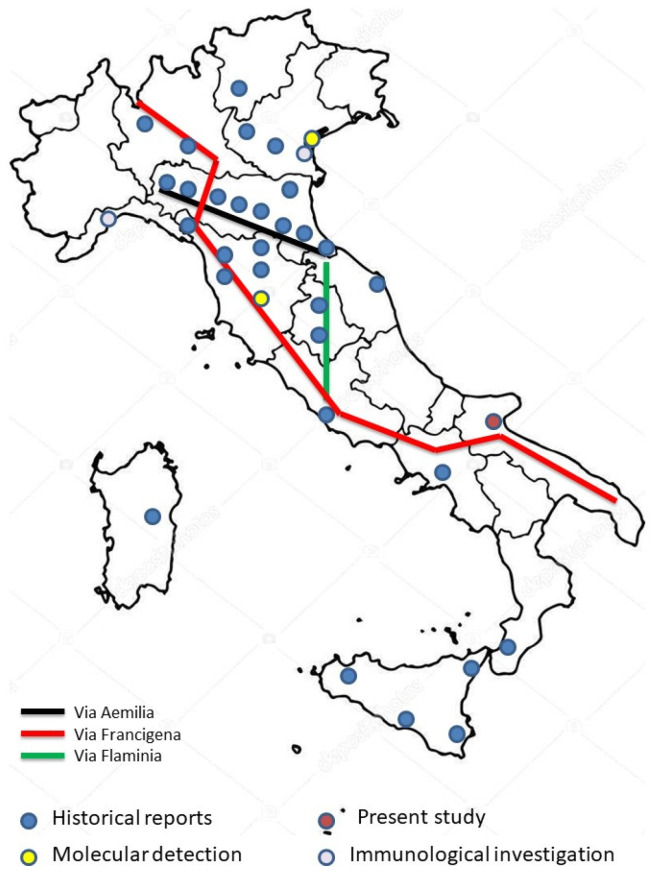
Geographical distribution of historical, molecular and immunological data related to Black Death reports in Italy.

Plague, caused by the bacterium *Yersinia pestis*, is the acute epidemic disease of rats and other wild rodents accidentally transmitted to humans mainly by the bite of infected fleas [59]; other blood-sucking ectoparasites, primarily body louse [60,61], are also involved in the human epidemic circle of the disease. This arthropod-borne disease, resulting in the most common clinical form, the bubonic plague, is characterized by painful, purulent lymphadenitis of one or more regional lymph nodes proximal to the portal of entry of the microbes [62]. The case fatality rate of the bubonic form is 40–70%. Plague infection can also be acquired through the manipulation, ingestion, and inhalation of aerosolized contaminated droplets from infected tissues, animals, or humans [63]. *Y. pestis* can be simply cultured and with a short incubation period, rapid onset and high fatality rate, consequently, it is considered a potential agent for bioterrorism purposes [64]. In the absence of prompt medical treatment, the pneumonic and septicemic forms cause death in 100% of those affected [65]. At present, plague is reported in Africa, the Middle East, Asia, and North and South America [66,67] with cases linked to flea infected bites and, also, to the consumption of raw or poorly cooked infected meat and the manipulation of contaminated animal carcasses [68,69]. Historically, *Y. pestis* has caused three major pandemics in human history; the “Black Death”, the beginning of the Second Plague Pandemic in Europe, was responsible for killing four million people from 1347 to 1351 [70]. In Italy, the Black Death spread when two Genoese galleys reached Messina in the second half of June 1347, and then Trapani, and crossed over Sardinia and Corsica to arrive in Genoa in the second half of July, and then in Lombardy, Tuscany, Marche, Sicily, Sardinia [71], Calabria [72] and Apulia [73]. The catastrophic effects of the Black Death’s arrival in Southern Italy are not well documented, and the majority of the graves discovered attributed to the Second Plague Pandemic are from the north [74,75]. Furthermore, the discovery of *Y. pestis* is only determined by molecular analysis in a few cases [76,77,78,79,80,81]. The route of the disease and the chronology of the infected cities were linked to the commercial trade services, but then the spread of the disease became more erratic, following travelers. In fact, the plague spread along the roads that connected Italy by sea and land (Figure 3). In this complex system of roads and movements, Siponto-Manfredonia [82,83] and the Abbey of San Leonardo occupied an important position. In fact, until the 13th century, Apulia was a central and strategic area for those headed to Jerusalem and the Holy Land. These are port cities equipped with facilities for the hospitality of the monastic knightly orders, such as in Barletta (Santo Sepolcro), in Siponto (San Leonardo), and in Terlizzi (Sovereto). Additionally, the internal routes—such as the Via *Francigena* that passed from Siponto and arrived at the Sanctuary of Monte Sant’Angelo—were crossed by many pilgrims who stopped to rest and recover in the typical institutions, the hospitals (*hospitali*), and the stations (*stationes*). When the abbey was entrusted to the Teutonic Order, whose rule had to assist people who were sick and those who were poor, the hospital was upgraded and emphasized. Unfortunately, between the 12th and 15th centuries, there are not many mentions of the hospital itself in San Leonardo and its activities in the documents. However, it is clear that the cemetery of the Abbey was not only reserved for the knightly order, and those who died in the hospital were buried in it. Moreover, Siponto-Manfredonia was also an important harbor [84], frequented by merchants and traders. In fact, in the port of Siponto, many goods were loaded and unloaded, which took the sea route toward the Mediterranean East and the Balkan side of the Adriatic or reached the markets of the kingdom [85,86,87,88]. Above all, the relationship with the food and wares produced in the hinterland was important. Several farms, some of which were owned by the ecclesiastical order such as the Teutonics of San Leonardo, were reported in accounting books [88]. Therefore, the presence of the port and the passage of galleys (particularly from Venice) for the transport of grain could have contributed to the spread of the plague as in other Italian and European cities (e.g., Messina, Marsiglia, etc.). The monastery is surrounded by the rivers Candelaro, Versantino and Pantano, which create swamps and are not far from the Adriatic beach of Siponto, dominated by the hot and humid Southern wind. Additionally, it is closed by the mountains, which prevent ventilation [10]. These diseases caused high mortality, such as that recorded, for example, in 1562, attributed to the “contagion of catarrhs” [89]. On the other hand, in San Leonardo in more recent times, deaths from fever, angina pectoris, trauma, and smallpox are also attested [11,59]. During and after the second pandemic, the word “contagion” began to only describe the plague and its person-to-person transmission by breath and touch in the medical treatises. While it is true that late medieval plague epidemics could occur at any time of the year, including January in inhospitable places, in the warmer Mediterranean, the Black Death and its recurrent attacks steadily peaked in the hottest and driest spots of the year, such as June and July. It is, therefore, not surprising that the hospital of San Leonardo was closed in the summer months [10] and may have been a place of contagion and spread during the medieval plague waves. Added to this is the fact that, given the rapid outcomes of the disease, it is not possible to trace lesions to the skeletal system, and so the paleopathological study conducted on individuals at the macroscopic level could not have provided useful research inputs. For these reasons, the presence of a large number of coins in these tombs—clearly positioned in hidden places among the clothes and not intentionally deposited—supported the hypothesis that the gravediggers did not want to touch and search the bodies for fear of contagion (for similar excavation contexts documented in Italy and Europe [90,91,92,93,94,95,96,97,98,99]. Hiding money, especially when traveling, was a common habit in the middle of the 15th century, with the custom of some patients not bringing money in a bag hanging from their waist but rather putting it in a handkerchief they used to wear. Sometimes, they even divided it into multiple containers (bags, or even handkerchiefs or cloth flaps); money was often sewn inside the fur or mantle. In some cases, they had the habit of holding a handbag with small change on the side to attract the attention of any brigands, hiding most of the money under their clothes [100]. Although it is likely that the cause of death of both individuals examined is linked to the clinical outcomes of the plague, documented through genetic analyses, it is evident that the individual of T.6 showed lesions attributable to an infectious pathology that debilitated him, probably limiting his range of motion of the shoulder and left upper limb, and necessitated some care. Given the multifocal dislocation of the pathological lesions, the reactive bone processes, the presence of diffuse cloacae connected to a pus-containing abscess, a preliminary diagnosis of suppurative osteomyelitis is hypothesized or, secondarily, septic arthritis [101,102]; these diagnoses can be further supported through specific analyses, but it is possible that they contributed to the physical weakening of the individual and his attack by the bacterium *Yersinia pestis*. At the same time, it is useful to remember that the individual from T.6 showed biomechanical indicators attributable to greater stress on the lower joints, data supporting the hypothesis that the subject carried out an activity in which long stretches of journeys were foreseen. Given the overall picture that has been possible to reconstruct through archaeological, numismatic, genetic, and anthropological data it, therefore, seems possible to hypothesize that the individual was either a merchant or a traveler parked at the San Leonardo structure. In this place, he could, in fact, have found support to be treated.

## 5. Conclusions

Siponto-Manfredonia was an important crossroads for humans and goods. First, some pilgrims crossed Italy along the roads that led from Rome to the nearby Sanctuary of Monte Sant’Angelo and embarked from the port of Siponto toward Jerusalem. The pilgrims stopped at the hospitals that also welcomed people who were poor and those who were sick, cared for by the knightly orders. Contrastingly, the city was certainly populated by merchants from the port who trafficked the goods produced in the area with the nearby Adriatic shore and the Holy Land. Places such as these were perfect centers for outbreaks and the spread of the plague. Unfortunately, Siponto-Manfredonia was also afflicted by the swamping of its coast and by an extremely hot climate, which generally facilitated the spread of diseases. The scenario, that two deceased people who were buried a short distance from each other (both in terms of place and, probably, of time) inside the necropolis of San Leonardo and kept clusters of coins on their person, has led us to hypothesize that it was not a mere coincidence, but rather the consequence of a fast and not too thorough preparation of the two corpses for burial due to the fear of contracting some form of contagion. Today, the results of DNA analysis allow us to confirm this hypothesis. In fact, the results of the molecular investigation corroborate the hypotheses made based on archaeological, taphonomical, and numismatic evidence and proof that the individuals exhumed from the cemetery of San Leonardo probably died of *Y. pestis* during the Black Death. This study has increased the knowledge of the Black Death in the south of Italy during the 14th century. In fact, the confirmation of the cause of death of these individuals from San Leonardo represents an important result because these graves are the first Black Death burials attested in Southern Italy for the 14th century. Another find is dated back to the 1656–1658 epidemic [103]. Additionally, identifying plague victims in San Leonardo highlights that the contamination progressively spread through principal communication routes. San Leonardo was not known to be a cemetery for plague victims, but the presence of a hospital for assistance of sick and destitute people made this function very plausible, similar to another case from Northern Italy [78]. In 1349, Manfredonia—the new Siponto—similar to the rest of Italy and the world, was struck by the Plague [104] and again in 1448 [6]. Therefore, it is extremely probable that some plague victims were hospitalized in San Leonardo [88]. Therefore, the burials investigated constitute material proof that the hospital and cemetery were fully active in those years. The overall study of the cemetery area, for which interdisciplinary methods of approach and more specific analyses are being defined, not only will increase the knowledge on depositional practices and biological profiles of individuals but also provide further evidence of the presence and the epidemiological environment of the pathogen.

## Figures and Tables

**Table 1:** Target genes, amplicon size and references used for pathogen species detection and internal DNA human control.

Target Organism	Target Gene	Expected Amplicon Size	Reference
*Brucella* spp.	* IS711 *	63 bp	Hinic et al., 2008 [50]
*Rickettsia* spp.	* glta *	167 bp	Mongkol et al., 2018 [51]
*Mycobacterium tubercolosis* complex	* mpb70 *	133 bp	Lorente-Leal et al., 2019 [52]
*Bartonella* spp.	* 16S/23S *	126 bp	Varagnol et al., 2009 [53]
* Yersinia pestis *	* pla *	144 bp	Stewart et al., 2008 [54]
	* glpD *	288 bp	Drancourt et al., 2007 [48]
*Plasmodium* spp.	* 18S rRNA *	189 bp	Laroche et al., 2017 [55]
Human genome	* β-Globulin *	110 bp	Greer et al., 1991 [56]

## Data Availability

All relevant data are provided in the manuscript. Raw data can be made available on reasonable demand.

## Supplementary Materials

The following are available online at https://www.mdpi.com/article/10.3390/pathogens10111354/s1, Figure S1: Map of participating labs: 1: LB1 in Manfredonia (FG); 2,3: Detailof Istituto Zooprofilattico Sperimentale dalla Puglia e della Basilicata in Foggia with locations of laboratories included in this project or discarded because potentially unsuitable caused high risk of cross contamination (Red star: Headquarters; Yellow spot: S.S. Ricerca e Sviluppo (LB2); DNA extraction; Blue spot: S.S. Virologia (LB3); DNA amplification; Black spot: Biotecnologia e Vaccini (this lab was discarded and it was not included in this project because it currently works on the molecular detection of Bioterrorism agents): 4: LB4, Vibrione Milano (Sequencing Center). Figure S2: A four-colour chromatogram showing the results of Sanger sequencing of *pla* gene detected in T.6 (A) e T.2 (B); Amplification plot of *pla* gene of *Yersinia pestis* using a FAM-dye revelation running on the StepOne Realtime PCR instrument (Applied Biosystem, ThermoFischer scientific, MA, USA); Nucleotide chromatogram of *Y.pestis glpD* gene sequenced from T.6 (D) and sequence alignment (E).
