# Supplementary material for: Genetic Evidence of the Black Death in the Abbey of San Leonardo (Apulia Region, Italy): Tracing the Cause of Death in Two Individuals Buried with Coins
Source: Pathogens. 2021 Oct 20;10(11):1354. doi: 10.3390/pathogens10111354 (PMC8619915; doi:10.3390/pathogens10111354)
Supplement: Supplementary file 1 [file pathogens-10-01354-s001.zip › pathogens-1371260-supplementary.pdf]

Supplementary data

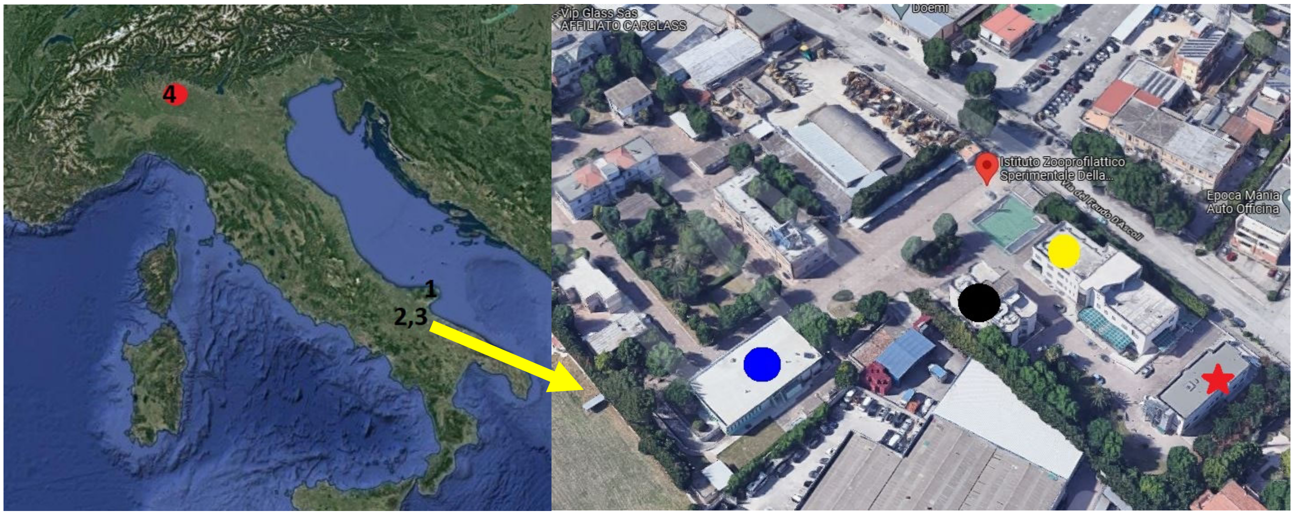

Figure S1.

Figure S1: Map of participating labs: 1: LB1 in Manfredonia (FG); 2,3: Detailof Istituto Zoopro-filattico Sperimentale dalla Puglia e della Basilicata in Foggia with locations of laboratories included in this project or discarded because potentially unsuitable caused high risk of cross contamination (Red star: Headquarters; Yellow spot: S.S. Ricerca e Sviluppo (LB2); DNA extraction; Blue spot: S.S. Virologia (LB3); DNA amplification; Black spot: Biotecnologia e Vaccini (this lab was discarded and it was not included in this project because it currently works on the molecular detection of Bioterrorism agents): 4: LB4, Vibrione Milano (Sequencing Center).

Figure S2.

A four-colour chromatogram showing the results of Sanger sequencing of *pla* gene detected in T.6 (A) e T.2 (B); Amplification plot of *pla* gene of *Yersinia pestis* using a FAM-dye revelation running on the StepOne Realtime PCR instrument (Applied Biosystem, ThermoFischer scientific, MA, USA); Nucleotide chromatogram of *Y.pestis glpD* gene sequenced from T.6 (D) and sequence alignment (E).

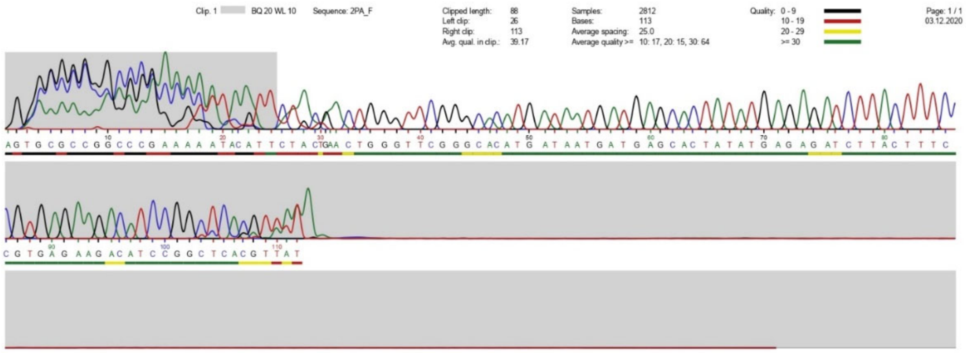

A

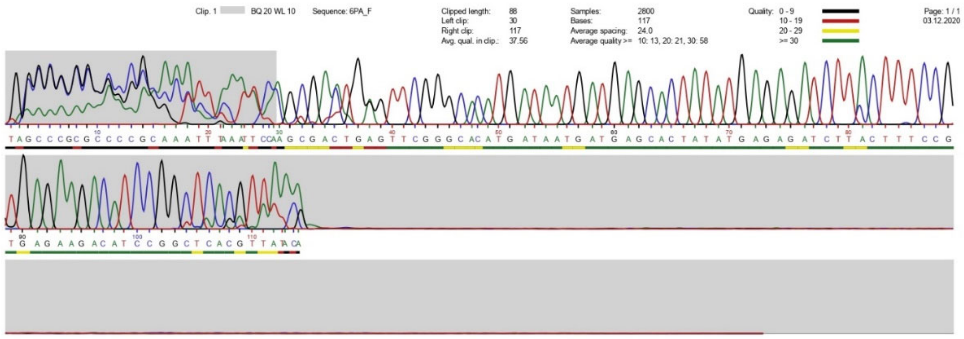

B

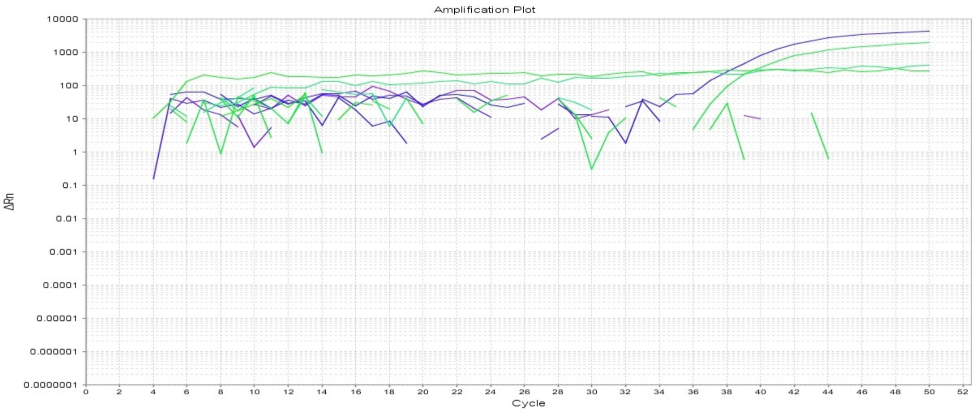

C

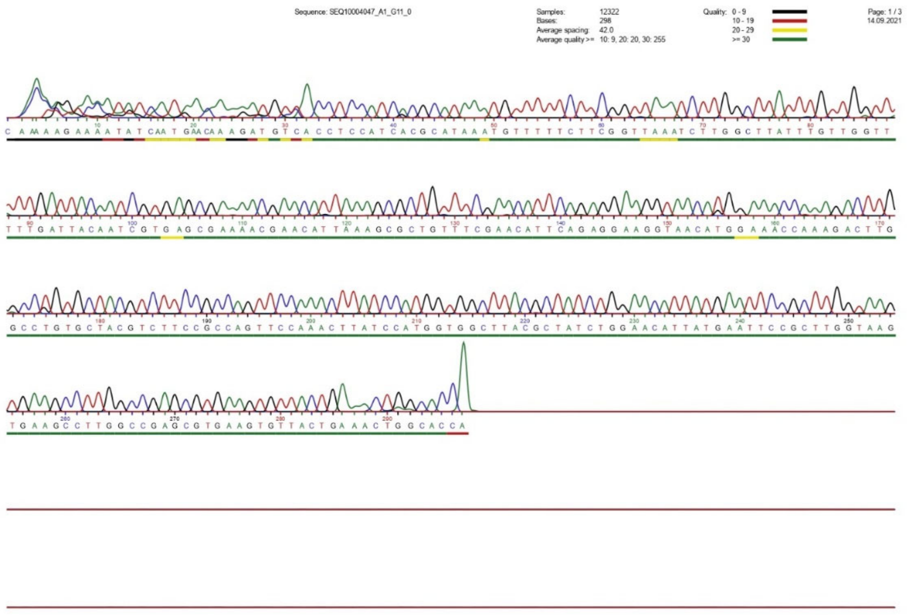
Sequence: SEQ10004047_A1_G11_0
Samples: 12322
Bases: 298
Average spacing: 42.0
Average quality >= 10: 9, 20: 20, 30: 255
Quality: 0 - 9
10 - 19
20 - 29
>= 30
Page: 1 / 3
14.09.2021

D

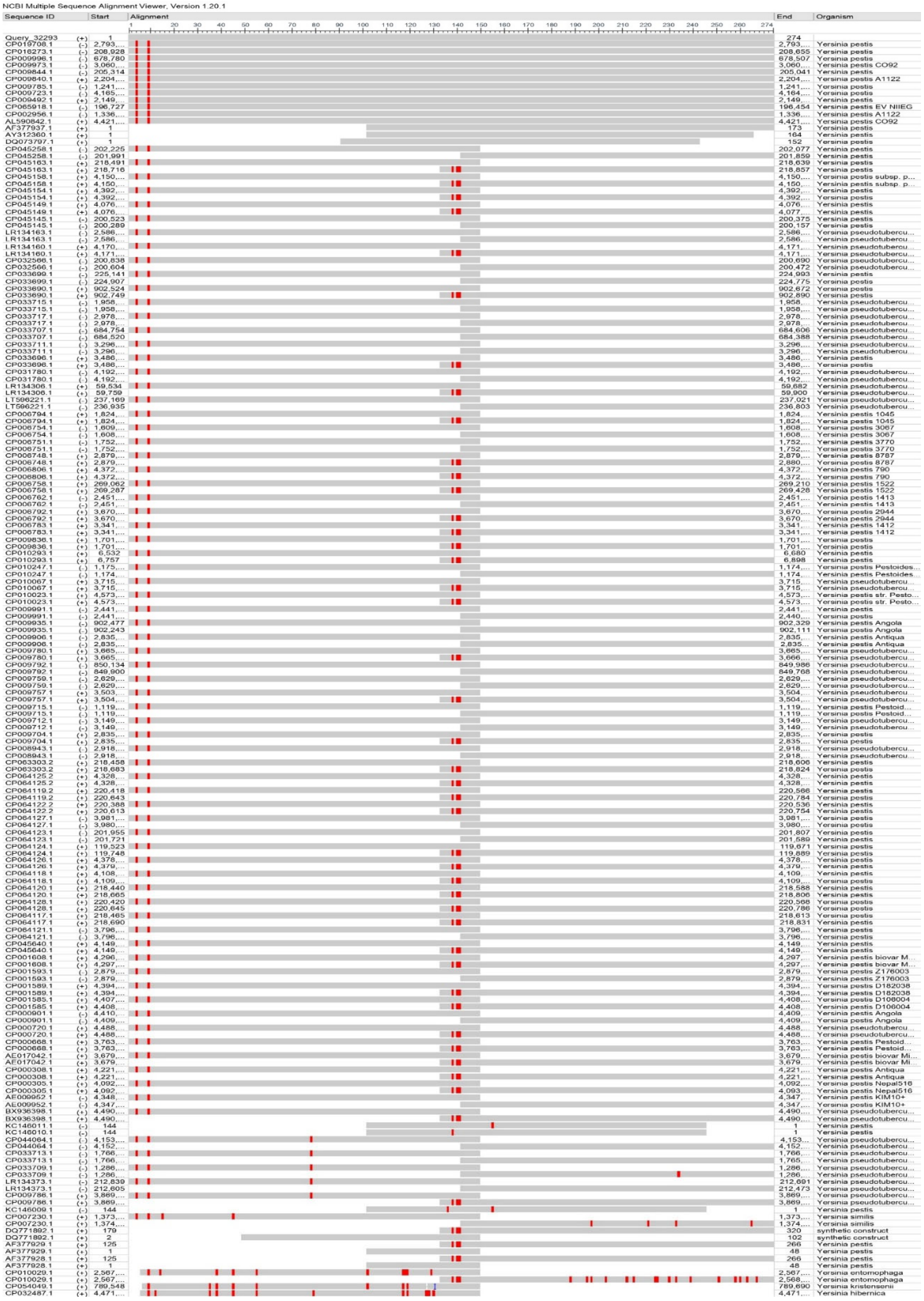
NCBI Multiple Sequence Alignment Viewer, Version 1.20.1
Sequence ID
Start
Alignment
End
Organism

E
